# Kaolin based protective barrier in municipal landfills against adverse chemo-mechanical loadings

**DOI:** 10.1038/s41598-021-89787-z

**Published:** 2021-05-14

**Authors:** Partha Das, Tadikonda Venkata Bharat

**Affiliations:** Indian Institute of Technology, Guwahati, Guwahati, 781039 India

**Keywords:** Environmental impact, Civil engineering

## Abstract

In this work, we assess the self-sealing and swelling ability of the compacted granular bentonite (GB) under an inorganic salt environment and induced overburden stresses from the landfill waste. The laboratory permeation tests with high ionic strength salt solutions reveal that the GB fails to seal and exhibits a significant mechanical collapse under different applied stresses. The applicability of GB in the form of geosynthetic clay liners as the bottom liner facilities in landfills that produce high ionic strength salt leachates, therefore, remains a serious concern. We propose an additional barrier system based on kaolin, for the first time, to address this problem. The proposed kaolin-GB layered system performs satisfactorily in terms of its sealing and swelling ability even in adverse saline conditions and low overburden stresses. The kaolin improves the osmotic efficiency of the self and also helps the underlying GB layer to seal the inter-granular voids. The estimated design parameters by through-diffusion test suggest that the kaolin-GB layered system effectively attenuates the permeant flux and suitable as a landfill liner.

## Introduction

Geosynthetic clay liners (GCLs) are the conventional liner material in municipal solid waste (MSW) landfills for inhibiting the migration of generated leachate to the environment. The economic viability and ease of handling, transportation, and installation are some of the virtues that replaced classical compacted clay liners with the GCL. The GCLs comprise a thin layer (~ 5 to 10 mm) of compacted granular bentonite (GB) in the form of uniform-sized granules sandwiched between two geotextile layers. The compacted GB contains large inter-granular voids at the natural air-dry state after placement in the field. These granules disintegrate upon wetting by the underlying native soil and swell into the inter-granular voids to seal the hydraulic pathways. The self-sealing ability of the GB^1–4^ also helps in sealing the technological gaps (i.e., puncture and loss of bentonite) created during the placement^5–7^. Thus, the saturated GCL acts as a hydraulic barrier due to very low hydraulic conductivity (K_s_ < 10^–9^ m/s)^8–10^. A slow diffusion process augmented by high sorption potential is the sole predominant mechanism for leachate transport through saturated GB in landfills when the hydraulic conductivity is ≤ 10^–9^ m/s^11^. Therefore, the sealing ability, low hydraulic conductivity, high sorption potential, and slow diffusion process make the GCL suitable for waste encapsulation in landfills. However, GB remains un-hydrated after the GCL placement during the exposure to the landfill leachates^12–14^. Thus the chemical compatibility of un-hydrated GB is receiving significant interest currently.

The MSW landfills generate leachate, which consists of an array of inorganic cations, viz., sodium (Na^+^), potassium (K^+^), and calcium (Ca^2+^), due to salt-laden solid waste from the rubber industry^15,16^; construction and demolition waste^17–20^; and inorganic hazardous waste from the electroplating industries^21,22^. Recent studies^3,23–25^ showed a significant reduction in the osmotic potential and manifold increase in the hydraulic conductivity of the GB in the presence of different high ionic strength inorganic salts. Lack of complete sealing of inter-granular voids in GB in the presence of high ionic strength salt solutions provides easy mobility of various contaminants, including viruses and pathogens, into the environment due to the current practice of virus-contaminated BMW disposal into the existing landfills^26–29^. However, the past studies^23–25^ do not consider the loading conditions imposed on the GCLs by the landfill wastes. The behaviour of GCLs due to exposure to such inorganic salt leachates under different overburden stresses arising from the waste load is vital for the overall hydraulic and mechanical stability of the landfills. These overburden stresses vary from low to high during the initial phase of waste disposal to the closure of the landfill^22,30,31^. The present work supported the past studies on GB's shortcoming for complete sealing upon permeation with high ionic strength salt solutions and under different overburden stresses. Thus identification of more chemically compatible material for safe waste management in landfills is the utmost pressing need.

**Figure 1:**
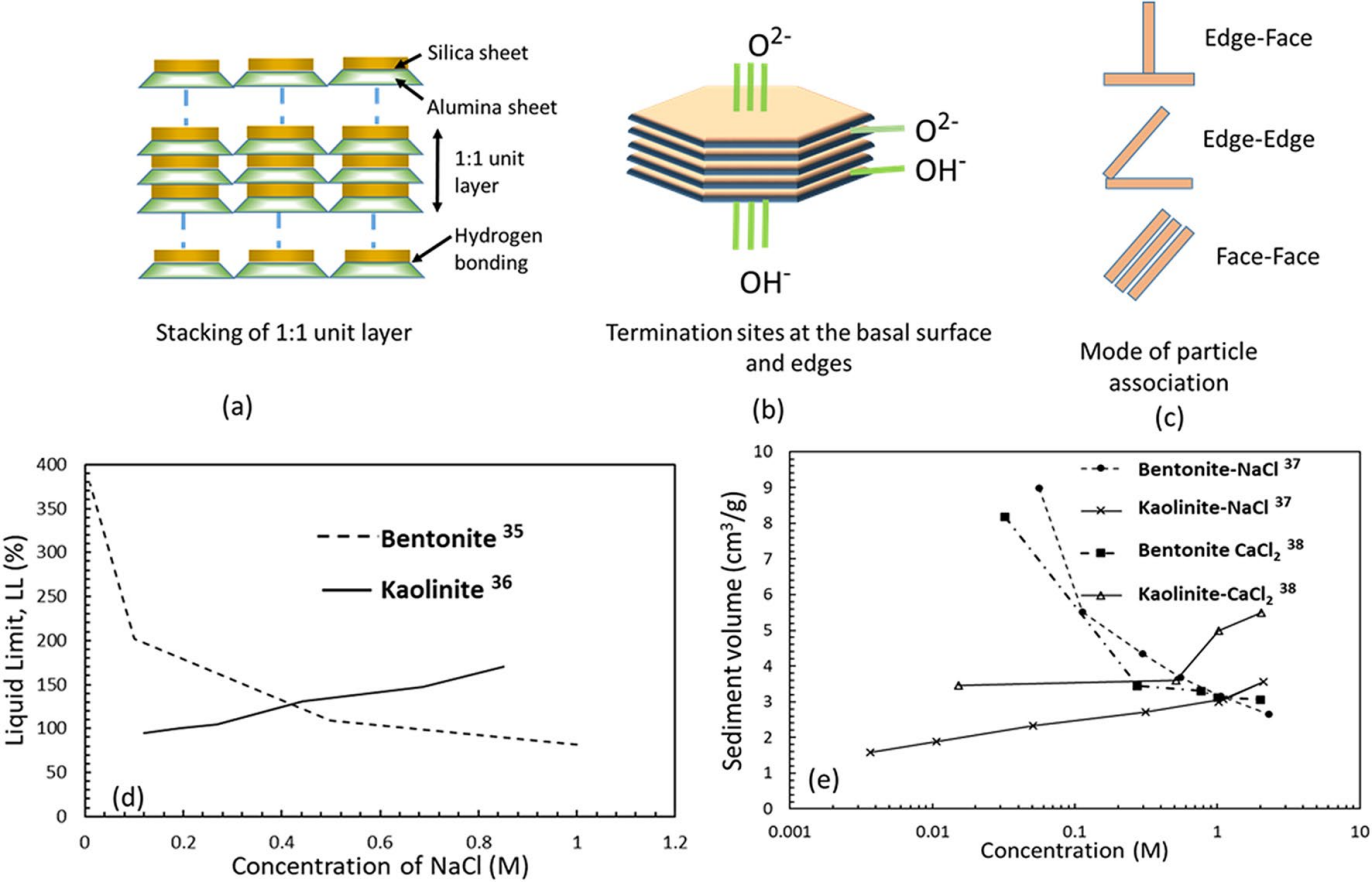
Illustration depicting the kaolinite structure (**a**) Stacking of unit layer, (**b**) termination sites, (**c**) modes of particle association, (**d**) variation of the liquid limit of bentonite and kaolinite with NaCl concentration, (**e**) variation of the sediment volume of bentonite and kaolinite with NaCl and CaCl_2_ concentration.

Kaolin is a prominent and natural clay soil, which is widely used in porcelain and cosmetic industries. This soil is rich in kaolinite mineral, which is formed by 1:1 stacking of tetrahedral silica sheets and octahedral alumina/gibbsite sheets (Fig. 1a). The silica sheet in kaolinite is comprised of Si^4+^ at the center and O^2−^ at the vertices. In comparison, the gibbsite sheet is formed of Al^3+^ at the center with OH^−^ at the vertices. This unique stacking of silica and alumina sheets by hydrogen bonding renders OH^−^ termination sites on the alumina face and O^2−^ termination sites on the silica face; and both OH^−^ and O^2−^ termination sites on the edges (Fig. 1b). These reactive termination sites in kaolinite mineral favor several modes of particle associations^32,33^ (Fig. 1c) due to the dominant surface forces. Most of the past studies provide the kaolin's chemical compatibility in the stress-free state, as summarized in Fig. 1d,e. The values of liquid limit and sediment volume of kaolin increase with salt concentration but decrease significantly for the bentonite^34–38^. Dispersed (face-face) fabric prevails between the individual particles in the presence of water or low concentration pore-fluids; in contrast, the flocculated (edge-face) structure prevails in high salt concentrations due to the reactive termination sites. The increased flocculation leads to swelling in kaolin at a stress-free state^38^. The chemo-mechanical loadings further control the magnitude of these inter-particle forces and the kaolin’s overall behavior. At lower initial compacted densities, kaolin exhibits significant collapse due to wetting^32^. Conversely, kaolin swells under low inundation stress with increased compaction effort^39^ due to the enhanced coulombic and van der Waals' attraction among the particles. The osmotic efficiency of compacted kaolin improves further in high ionic strength inorganic salt solutions^32^.

Thus kaolin exhibits strikingly contrasting behaviour in comparison to the bentonite in the presence of different pore-fluids and compaction densities. Further, the liquid limit and sediment volume are surrogate compatibility indices to predict the long-term hydraulic behaviour of the clays^34^. The improvement in the osmotic efficiency and chemical compatibility of kaolin is expected to favor the reduction in hydraulic conductivity in the presence of a high ionic strength salt environment in the landfills. Thus compacted kaolin might be helpful to restrict the leachate migration in the landfills under high ionic strength saline conditions. In contrast, the GB alone struggles to seal the inter-granular voids under such chemical loads. However, no study is available to explore the chemo-mechanical behaviour of kaolin under landfill conditions.

In this work, the inter-granular sealing behaviour of GB exhumed from the commercial GCL was studied under the salt environment and under two different overburden stresses in permeameter cells. The self-sealing ability of GB was completely lost, and significant volumetric collapse was observed upon permeation with high ionic strength salt solutions and under low overburden stress. The osmotic efficiency of commercial kaolin under such a salt environment was explored for the first time. The chemical compatibility of compacted kaolin was promising when paired with GB under extreme saline conditions and favored the GB to completely seal and significantly reduce the volumetric collapse. The underlying mechanisms for the sealing and swelling behaviour of GB and kaolin under the studied conditions were analyzed using the microstructural investigation. The laboratory through-diffusion tests also showed that the kaolin-GB layered system outperformed the individual GB layer in terms of contaminant migration rate and sorption potential.

## Results

**Figure 2:**
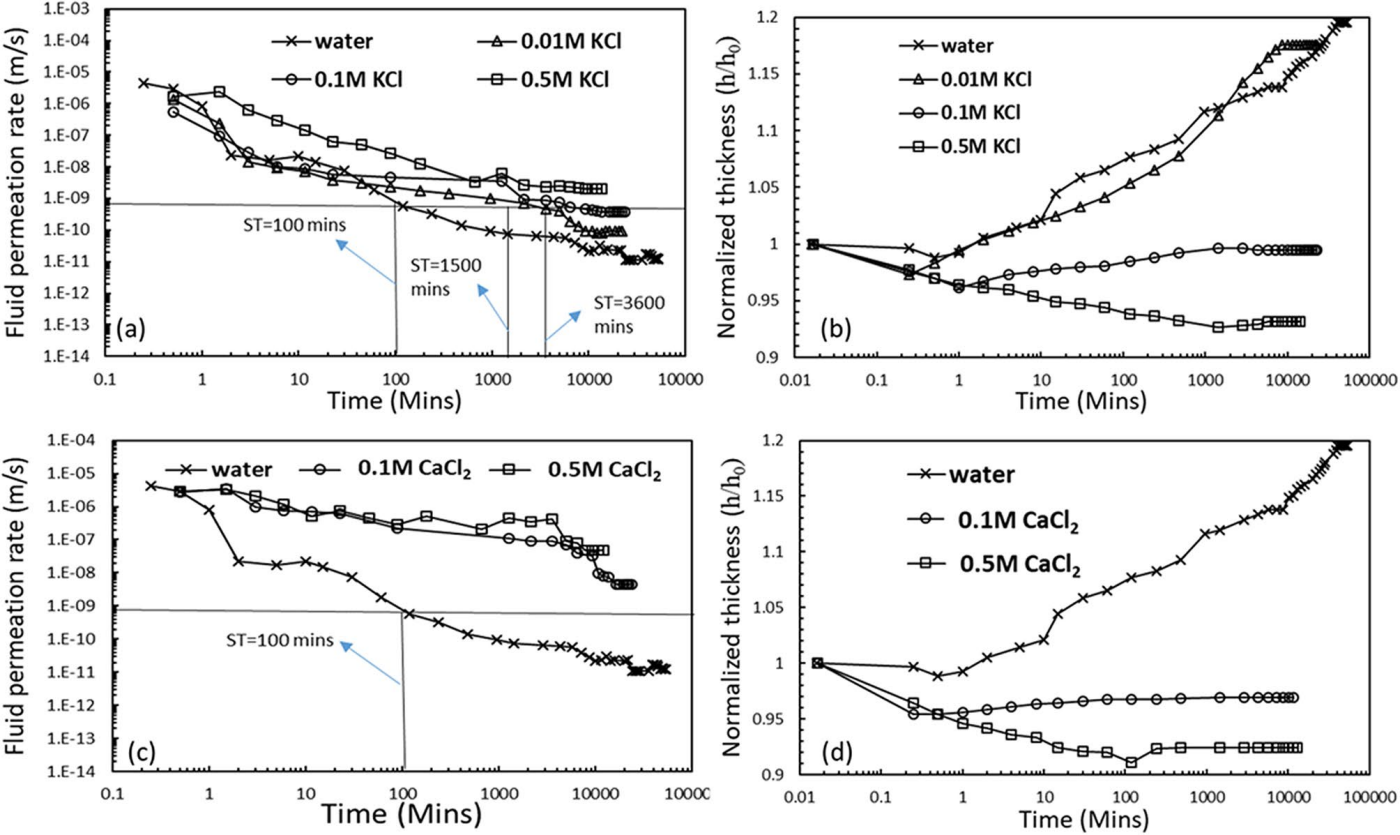
Temporal variation of (**a**) fluid permeation rate, and (**b**) normalized thickness for the GB under applied stress of 50 kPa in the presence of water and various concentrations of KCl; temporal variation of the (**a**) fluid permeation rate, and (**b**) normalized thickness of GB under applied stress of 50 kPa in the presence of water and various concentrations of CaCl_2_.

The temporal variation of permeation rate of water and different ionic strengths of KCl through compacted GB under the applied stress of 50 kPa was presented in Fig. 2a. The permeation rate of water through GB was very high in the initial 30 min, but the rate significantly dropped after 90–100 min due to the sealing of the inter-granular voids in GB. On the other hand, the estimated sealing time (ST) for the GB in the presence of 0.01 M and 0.1 M KCl was 1500 min and 3600 min, respectively. Further, complete sealing of the inter-granular voids could not be achieved in the presence of 0.5 M KCl, and the measured fluid permeation rate was higher than the limiting value (1 × 10^–9^ m/s) even after the equilibrium. The corresponding volume change during the pore-fluid permeation was presented in terms of temporal variation of the normalized thickness (*h/h*_0_) of the GB specimen in Fig. 2b. Normalized thicknesses of 1.2 and 1.1.7 were observed for GB at equilibrium due to the permeation of water and 0.01 M KCl solution, respectively, which indicates appreciable swell in GB after the initial collapse. However, the swelling was minimal in the presence of 0.1 M KCl with an equilibrium normalized thickness of ~ 0.99. Moreover, the GB in the presence of 0.5 M KCl experienced significant mechanical collapse for a large duration, and a marginal swelling was observed only after 2000 min. A normalized thickness of 0.95 for GB with 0.5 M KCl was observed at the time of test termination (Fig. 1b).

The detrimental effect of CaCl_2_ at 50 kPa mechanical stress was observed on the sealing ability and volume change, as shown in Fig. 2c. The compacted GB could not seal the inter-granular voids even at 0.1 M CaCl_2_ concentration, and the equilibrium hydraulic conductivity was an order of magnitude higher than the limiting value. The equilibrium conductivity increased a magnitude further with the 0.5 M concentration. The mechanical collapse was also significantly high due to the permeation of 0.1 M and 0.5 M CaCl_2_ solutions (Fig. 2d). Thus, the normalized thickness for the GB at the time of termination of the tests was found to be the lowest. A similar trend of fluid permeation rate and variation in the normalized thickness was exhibited by the GB in the presence of different concentrations of KCl and CaCl_2_ under the mechanical stress of 100 kPa. However, the ST of the GB improved, and the limiting value of 1 × 10^–9^ m/s was achieved at a lesser time in comparison to lower mechanical stress. Moreover, the swelling was significantly reduced under 100 kPa mechanical stress, and collapse behaviour was more predominant in the presence of all the studied concentrations of KCl and CaCl_2_, in particular at 0.1–0.5 M concentrations (Fig. S1 Supplementary Information).

**Figure 3:**
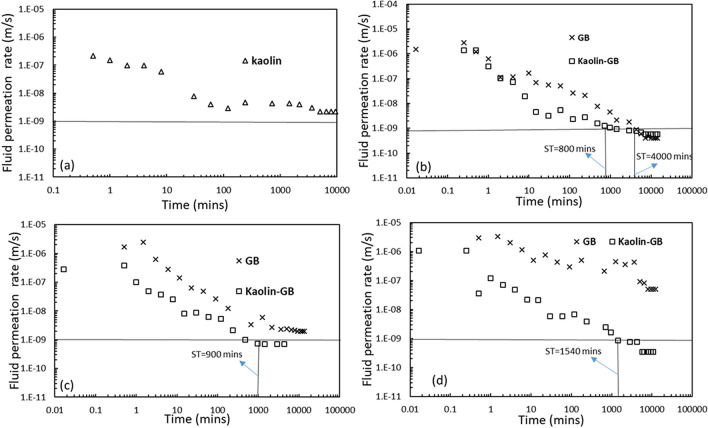
Temporal variation of fluid permeation rate through individual kaolin layer under applied stress of 50 kPa, comparison of the temporal variation of the fluid permeation rate for the GB and Kaolin-GB layered system in the presence of 0.5 M concentration of (**a**) NaCl; (**b**) KCl, (**c**) CaCl_2_ under applied stress of 50 kPa.

**Figure 4:**
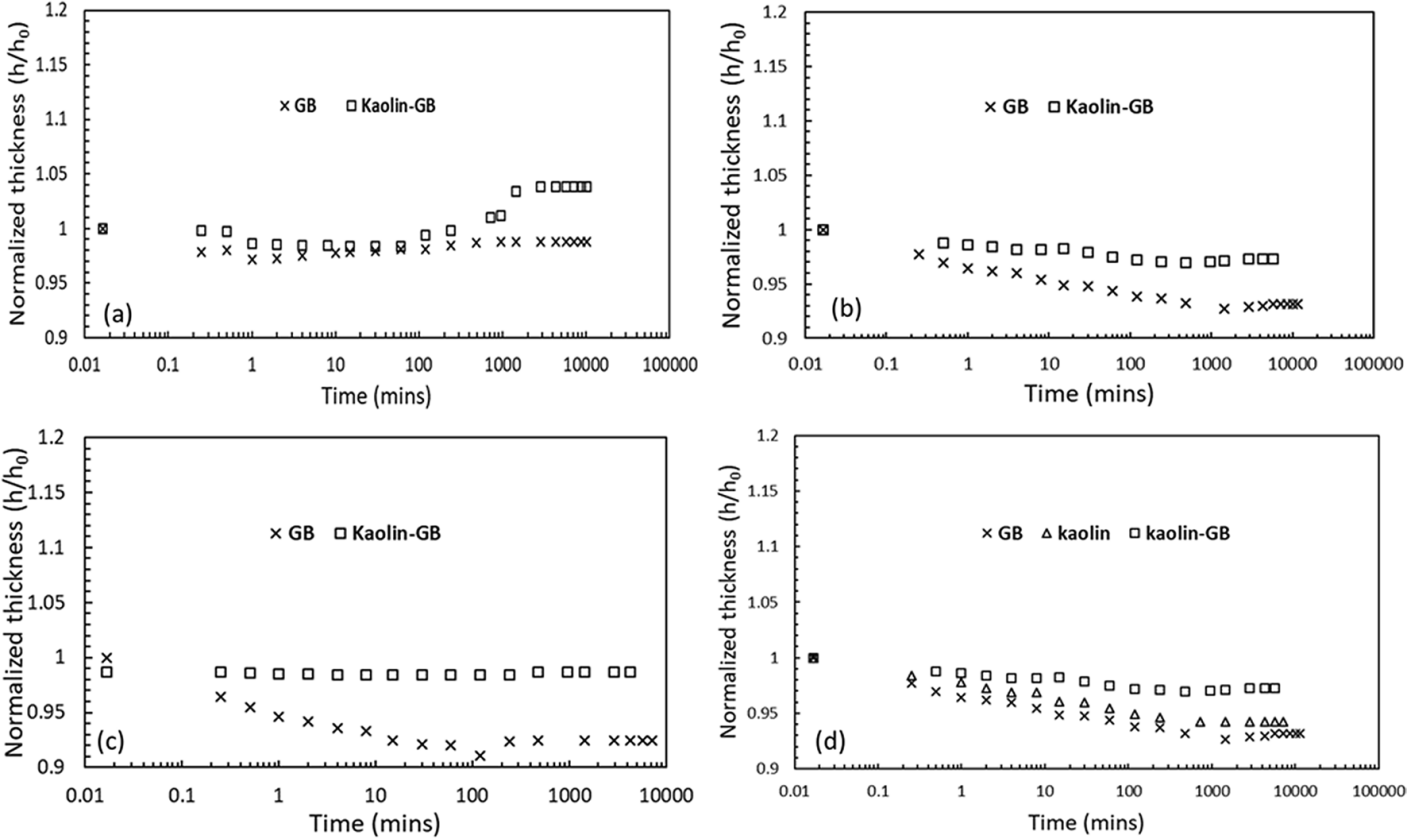
Comparison of the temporal variation of the normalized thickness for the GB and kaolin-GB layered system in the presence of 0.5 M concentration of (**a**) NaCl; (**b**) KCl, (**c**) CaCl_2_ under applied stress of 50 kPa; comparison of the normalized thickness of the GB, kaolin, the kaolin-GB layered system in the presence of 0.5 M KCl under 50 kPa stress.

A poor performance of GB in terms of sealing and swelling ability was observed in the presence of high ionic strength solution (*n* = 0.5 M) of a given salt and under 50 kPa applied stress. The inability of GB to seal in the presence of high ionic strength salt solution has the potential to provide easy access to various other contaminants present in the landfill leachate. Further, the excessive collapse of the liner materials could lead to mechanical instability of the landfill and result in a fatal consequence. In order to address this problem, kaolin was explored to improve the sealing ability by enhancing the osmotic efficiency in a high saline environment. Owing to the contrasting behaviour of kaolinite and bentonite in the presence of various pore-fluids (Fig. 1d,e), the sealing ability and volume change characteristics of the kaolin layer were presented in Figs. 3 and 4.

The fluid permeation kinetics through an individual kaolin layer in the presence of 0.5 M KCl and at 50 kPa applied stress was presented in Fig. 3a. The fluid permeation rate decreased with time, and the equilibrium conductivity reached ~ 3 × 10^–9^ m/s, which was slightly higher than the limiting value (1 × 10^–9^ m/s). Although a significant improvement in the conductivity of the kaolin was noticed, the kaolin layer was not able to maintain a fluid permeation rate below the limiting value. Thus individual kaolin fails to completely seal the hydraulic paths due to permeation with high ionic strength salts. The temporal variation of the permeation rate through the kaolin-GB layered system in the presence of high ionic strength (*n* = 0.5 M) solutions of NaCl, KCl, and CaCl_2_ at 50 kPa stress was presented in Fig. 3b–d. The performance of the individual GB layer under the same conditions was also presented in the same figure for drawing a comparison. The ST reduced considerably for the kaolin-GB layered system in comparison to the individual GB layer for any given salt solution. The individual GB layer took ~ 4000 min to seal in the presence of 0.5 M NaCl solution, but the kaolin-GB layered system sealed in 800 min (Fig. 3b). Similarly, the kaolin-GB layer took ~ 900 min and 1540 min to seal the inter-granular voids in the presence of 0.5 M KCl and CaCl_2_, respectively, while the individual GB layer could not seal under the same conditions (Fig. 3c,d).

Further, the temporal variation of the normalized thickness of individual GB layer and the kaolin-GB layered system was presented in Fig. 4a–c. In the presence of 0.5 M NaCl solution, the kaolin-GB layer showed a considerable amount of swelling after a period of ~ 200 min, while the individual GB did not swell after the initial collapse (Fig. 4a). Moreover, the kaolin-GB layered system showed relatively a lesser collapse, and slight swelling was observed after ~ 500 min with an equilibrium normalized thickness of ~ 1 in the presence of 0.5 M KCl and CaCl_2_ (Fig. 4b,c). However, the individual GB layer exhibited mechanical collapse for a large duration, and the equilibrium thickness at the time of termination was 0.95 and 0.93 in the presence of 0.5 M KCl and CaCl_2_, respectively, under the same conditions. The variation of the normalized thickness of the individual kaolin layer indicated that at higher salt concentration, the kaolin exhibits lesser collapse in comparison to the GB layer, however, with negligible swelling. On the other hand, the kaolin-GB layered system exhibits minimal collapse under similar conditions (Fig. 4d).

**Figure 5:**
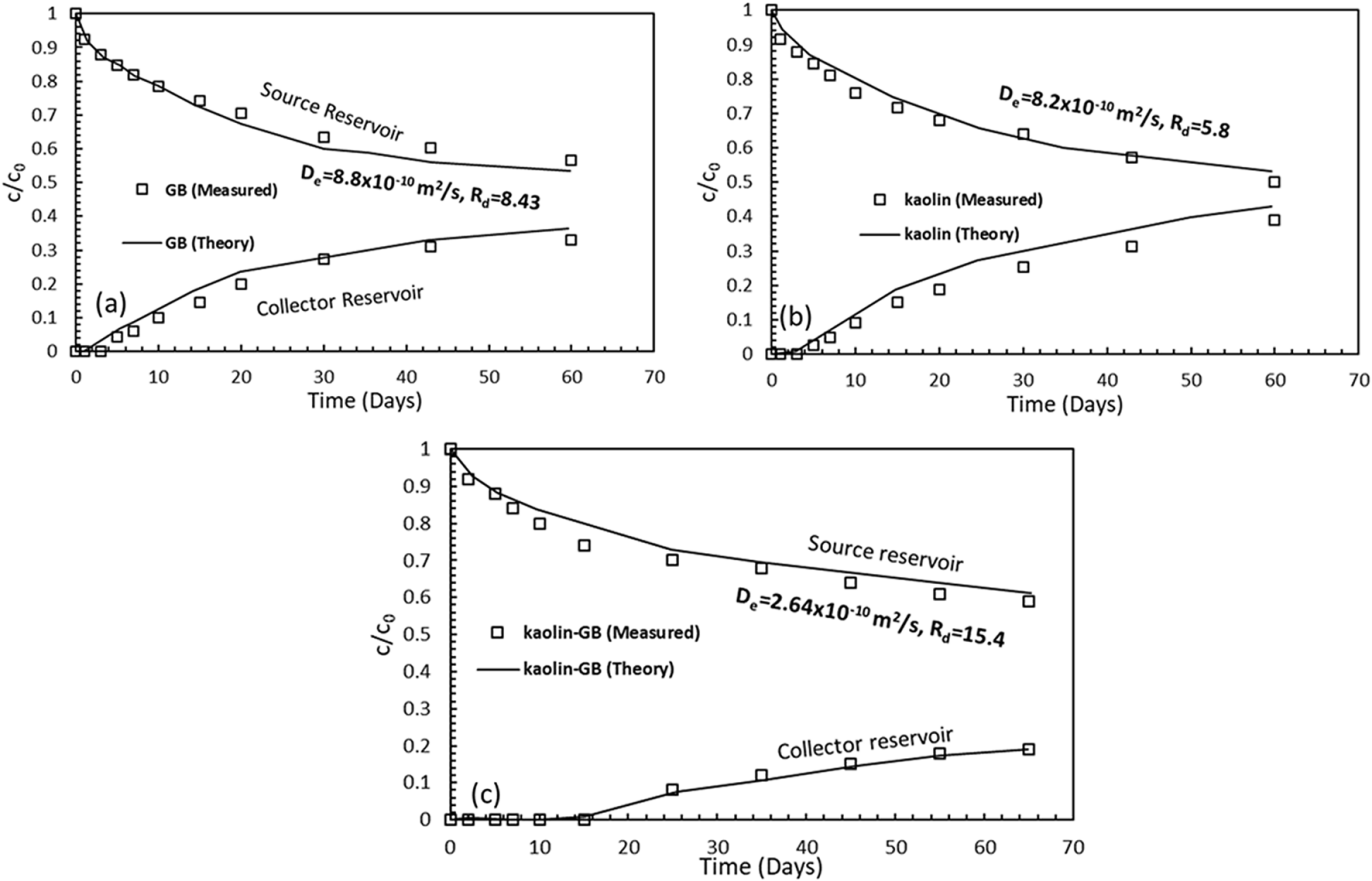
Comparison of the measured and theoretical concentration data in the presence of 0.5 M KCl for the (**a**) GB layer, (**b**) kaolin layer, and (**c**) kaolin-GB layered system.

Diffusion will be the dominant transport mechanism in the kaolin-GB layered system as the proposed combination exhibited good sealing ability and required saturated conductivity in the presence of high ionic strength salt solutions. Although the individual GB and kaolin could not achieve the limiting hydraulic conductivity and the hydraulic transport is the dominant mechanism, the diffusion characteristics were presented along with the kaolin-GB layered system for the sake of comparison. The diffusion characteristics of GB, kaolin, and kaolin-GB layered system using the critical case, i.e., 0.5 M KCl solution in the source reservoir, were presented in Fig. 5a–c, respectively. The measured salt concentration data in the source and collector reservoirs were expressed as relative concentration (*c/c*_0_) by normalizing the solute concentration at any time interval, *c*, with the initial concentration, *c*_0._ The *c/c*_0_ in the collector reservoir at the end of 60 days for the kaolin-GB layer was ~ 0.2; on the other hand, the *c/c*_0_ in the collector reservoir for individual GB and kaolin layer was ~ 0.38, indicating lesser diffusive flux through the kaolin-GB layer. The diffusion rate of potassium ion through the kaolin-GB layered system was slowest among the studied cases. The effective diffusion coefficient (*D*_*e*_) and retardation factor (*R*_*d*_) were estimated by minimizing the error between the theoretical and measured temporal concentration profiles^40,41^. The theoretical plots were in good agreement with the experimental data. The estimated theoretical concentration curves generated in the source and collector reservoir using the optimized model parameters were also presented in Fig. 5a–c. The *D*_*e*_ was lowest, and sorption potential was highest for the kaolin-GB layered system among the studied cases indicating the superior performance of the proposed kaolin-GB layered system for the potential liner in the MSW landfills.

## Microstructural analysis

**Figure 6:**
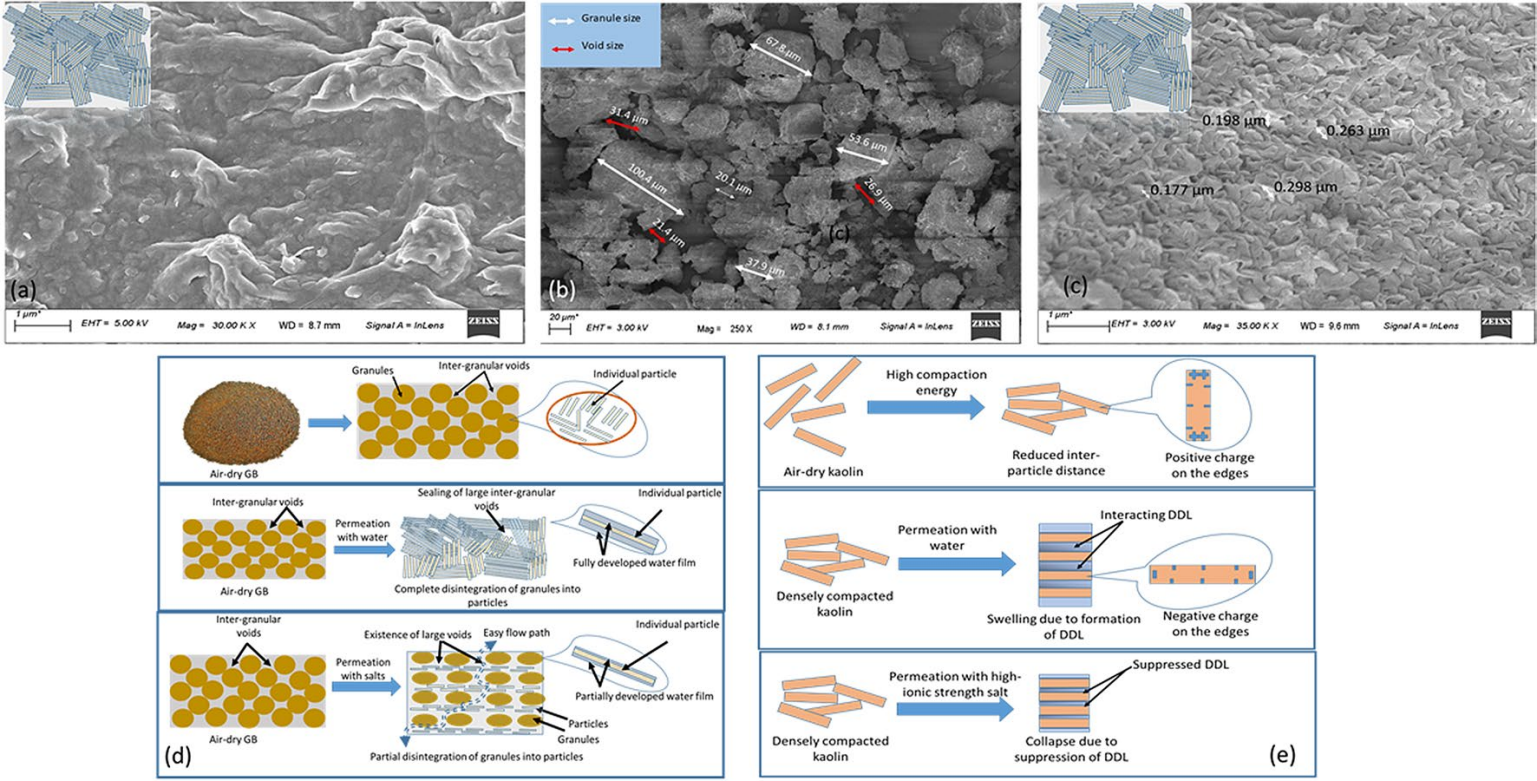
FESEM image of GB as individual layer (**a**) in the presence of water, (**b**) in the presence of 0.5 M KCl, (**c**) FESEM image of the GB of the kaolin-GB layered system upon permeation with 0.5 M KCl, (**d**) illustration depicting complete and partial disintegration of the granules into particles in the presence of water and salts, respectively (**e**) particle orientation of kaolin in the presence of water and high-ionic strength salt solution.

The type and concentration of the permeating fluid played a significant role in influencing the sealing and swelling ability of the studied GB. The experimental results revealed that the granules of GB fail to swell appreciably and seal the inter-granular voids in the presence of high ionic strength (*n* = 0.5 M) salt solutions and different applied mechanical stresses. The microstructure of GB at equilibrium under different chemo-mechanical loadings was studied using the Field Emission Scanning Electron Microscope (FESEM). The specimens after the inundation were obtained from the permeameter with a minimal disturbance. The specimens were Lyophilized (FreezeDry System, LabconcoFreeZone) at − 60 °C to preserve the specimen's fabric during the testing. The FESEM image of the saturated GB layer in equilibrium with water and 0.5 M KCl under 50 kPa applied stress was shown in Fig. 6a,b, respectively. The water-saturated GB showed (Fig. 6a) complete closure of pores due to fully expanded mineral sheets. On the other hand, the saturated GB with 0.5 M KCl concentration was characterized by 20–100 μm size granules and ~ 20 to 30 μm size inter-granular voids (Fig. 6b). Therefore, the inability of the large-sized granules to disintegrate into individual particles was understood to be the primary reason behind the poor sealing and swelling ability of GB in the presence of high ionic strength salt solutions. The experimental results from the present study also revealed that the ST reduced, and the GB performance improved when the tests were conducted at 100 kPa mechanical stress. The improvement was due to the assistance of higher mechanical stress in disintegrating the granules. However, the mechanical collapse increased at 100 kPa mechanical load in the presence of a high ionic strength solution of KCl and CaCl_2_ solutions. The increase in collapse is primarily due to the swelling pressure of the GB in the presence of a high ionic strength solution of KCl and CaCl_2_ is lesser than the applied stresses.

The fluid permeation rate through the individual kaolin layer in the presence of 0.5 M KCl decreased with time, although the limiting value of 1 × 10^–9^ m/s could not be achieved under the lower applied stress (50 kPa). On the other hand, under similar loading conditions, the limiting value was achieved for the kaolin-GB layered system in the presence of high ionic strength solution of NaCl, KCl, and CaCl_2_. The FESEM image of the GB in the kaolin-GB layered system after equilibration with 0.5 M KCl indicates that the granules disintegrated into individual particles of the size range of 0.17–0.26 μm (Fig. 6c). Thus the sealing ability of the GB in the kaolin-GB layered system in the presence of 0.5 M KCl is comparable to the individual GB layer in the presence of water or lower salt concentrations. Elemental analysis of different layers upon permeation with 0.5 M KCl was undertaken to understand the effect of kaolin inclusion on the sealing ability of adjacent GB layer under high ionic strength salt solutions.

**Figure 7:**
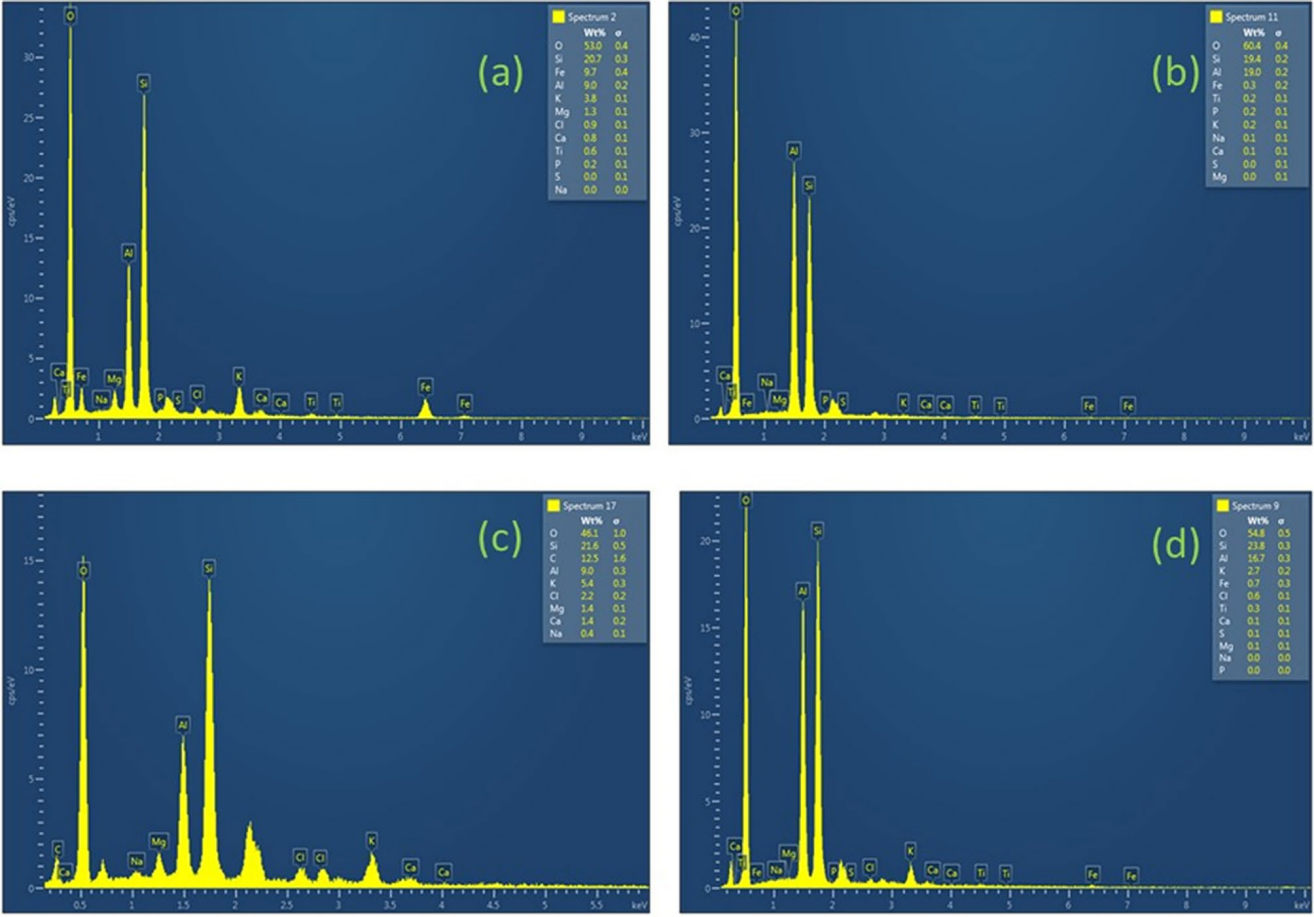
EDX spectra of (**a**) kaolin sample in the kaolin-GB layered system post equilibration with 0.5 M KCl, (**b**) kaolin sample in the natural condition, (**c**) Individual GB layer post permeation with 0.5 M KCl, and (**d**) GB sample of the kaolin-GB layer post permeation with 0.5 M KCl.

The adsorption potential of kaolin for the potassium salts was analyzed by the energy-dispersive X-ray (EDX) spectroscopy for the kaolin sample present in the kaolin-GB layered system after equilibration with 0.5 M KCl. The EDX enables identifying the elemental composition in a selected spectrum (micro-area) of the tested sample. The EDX spectra of the kaolin sample in the proposed kaolin-GB layered system were presented in Fig. 7a. The percentage range of various elements in the kaolin sample was analyzed in the adequate voltage range as presented. Apart from the structural elements, the kaolin in the kaolin-GB layer exhibited a high peak for potassium (K^+^) with a weighted percentage of 3.8. On the other hand, the EDX spectra of the kaolin sample in its natural condition showed a negligible peak for potassium with a weight percentage of 0.2 (Fig. 7b). However, the spectra showed an excellent adsorption affinity of kaolin for K^+^ ions upon permeation with 0.5 M KCl solution. Moreover, a very high peak for potassium with a weight percentage of 5.4 was found for the individual GB layer after permeation with 0.5 M KCl. The weight percentage of potassium for the GB sample of the kaolin-GB layer reduced under a similar condition to 2.7 due to exposure to a lower concentration of salts in the GB layer (Fig. 7c,d). The EDX results, therefore, confirm that the kaolin in the kaolin-GB layered system adsorbed a significant amount of potassium in the presence of 0.5 M KCl. Complete closure of the inter-granular voids enables the GB layer to allow only diffusion mechanism for the ion transport.

## Discussion

Surface forces control the behaviour of clays under chemo-mechanical loading. The van der Waals forces tightly hold individual particles of bentonite in the GB at the dry state. The hydration of clay particles by the permeating water and lower ionic strength salts generate repulsive (osmotic) forces between the hydrated particles^42–45^. The granules disintegrate into individual particles when the developed osmotic forces overcome the van der Waals attraction forces. Consequently, the hydrated clay particles swell into the inter-granular voids and restrict the hydraulic pathways to maintain a hydraulic conductivity < 10^–9^ m/s. Further, the specimen exhibits macroscopic swelling once the inter-granular and inter-aggregate voids seal completely due to significant osmotic pressures (Fig. 6d). The increase in the ionic strength of the salts decreases the thickness of diffuse double layers (DDLs) significantly. Thus the reduced osmotic pressures between the hydrated particles could not overcome the van der Waals forces to completely disintegrate the granules, as evidently seen in Fig. 6b.

The mechanical load during the pore-fluid inundation helps in the disintegration of the granules against the van der Waals' attraction. The sealing ability thus improved with the increase in the mechanical load. However, the swelling pressure of the bentonite decreases with the increase in the ionic strength of the salts as the osmotic pressures reduce^46^. Bentonite subsequently exhibits a mechanical collapse if the swelling pressure is smaller than the applied mechanical stress. The GB exhibits significant collapse with higher applied load and high ionic strength salts in the present work. A significant mechanical collapse is unfavorable for the mechanical stability of the landfill. Similarly, an incomplete sealing of the inter-granular voids results in the development of large hydraulic gradients and pore pressures that affect overall hydraulic and mechanical stability.

The present study shows an overall improvement in the sealing and volume change behaviour due to the placement of the kaolin layer over the GB. The combination of coulombic and van der Waals' forces predominantly influences the compacted kaolin behaviour under chemo-mechanical loadings. The higher compaction effort at air-dry kaolin significantly increases the attractive forces while reducing the inter-particle distances. Kaolin hydration by water alters the positive charges to negative on the particle edges and nullifies the coulombic attraction between the basal face–edges^32^. A DDLs formation around the hydrated kaolinite particles in densely compacted kaolin results in osmotic pressure development and subsequent macroscopic swelling (Fig. 6e). The presence of high ionic strength salts suppress the diffuse double layer thickness similar to GB and exhibited slight collapse. However, the observed collapse is smaller due to smaller inter-particle distances and thinner DDLs due to smaller surface charge density of kaolinite, as evident from the smaller equilibrium hydraulic conductivity (~ 3 × 10^–9^ m/s) for kaolin alone. Moreover, the saturated kaolin retains a significant amount of cations. The adsorption of monovalent and divalent cations to form inner-sphere complexes occurs mostly on kaolin's edges due to the presence of silanols and aluminum hydroxyls^47–49^. The ion exchange between the cations in the bulk solution and the hydroxyl functional groups on the edges further leads to interface polarisation^33^. These mechanisms allow only reduced concentration of the salt permeants into the underlying GB layer to help in sealing the inter-granular voids entirely and achieve the limiting hydraulic conductivity. The sealing of GB further reduces collapse potential and makes it suitable in landfills for preventing the hydro-mechanical instabilities. Additionally, chemical diffusion would be the dominant transport mechanism through the proposed barrier system.

The through-diffusion test results reveal that the proposed kaolin-GB layer reduces the diffusive flux into the surrounding geology. The sorption and the interface polarisation ability of kaolin aided by the smaller inter-particle distances increase the tortuous pathways through the kaolin-GB layered system. Further, the thicker DDLs around the bentonite particles due to exposure to lower chemical concentrations improved the tortuous paths. The proposed combination of liner thus provides the lowest value of diffusion coefficient and highest retardation factor. The introduction of naturally available kaolin clay in landfills for the bottom liner facility combined with existing GCLs addresses the problem of hydro-chemo-mechanical instabilities due to municipal landfill leachates.

## Materials and methods

### Materials

Granular bentonite in the present study was exhumed from the commercial GCL (Maccaferri, India) used for landfill applications. The GB mass per unit area was 3.96 kg/m^2^ and a thickness of ~ 5 mm. The average granules size was ~ 0.7 mm. The GB used in the present work was considered to be one of the most suitable liner materials for MSW landfills based on the index and surface properties that were presented in Table 1. The specific gravity of the GB was determined by the density bottle method as per IS-2720-3 (Part 3)^50^. The liquid limit and the plastic limit of the GB were determined as per the standard IS-2720-5 (Part 5)^51^, and the shrinkage limit was obtained by following the procedure as per IS-2720-6 (Part 6)^52^. The total cation exchange capacity (CEC) was estimated based on the standard procedure^53^, and the specific surface area (SSA) was determined using ethylene glycol mono-ethyl ether (EGME) procedure on four duplicate samples^54^. Further, a commercially available kaolin clay without any pre-treatment was also used in the present study to assess the applicability of the kaolin for the barrier application in landfills. The basic properties of kaolin based on the recent study by Choudhury and Bharat^32^ were reproduced in Table 1.

**Table 1:** Index and surface properties of the studied clays.

Property	GB	Commercial kaolin^32^
Specific gravity	2.75	2.62
Liquid limit	458	40
Plastic limit	56	32
Shrinkage limit	15	28
Total CEC (meq/100 g)	97.9	5
Specific surface area (SSA)	505	12

### Permeant fluids

Distilled water and chloride salt solutions comprising sodium, potassium, and calcium were used as pore-fluids to study the sealing and macroscopic swelling potential of the compacted GB, kaolin, and kaolin-GB layered system due to salt permeation. The analytical grade chemicals corresponding to a purity of 99% were procured from Spectrochem (India), and supplied by a local vendor. The tests were performed at three distinct concentrations, viz., 0.01, 0.1, and 0.5 M for the individual salts by dissolving the salt of the required mass in distilled water at room temperature.

### Methodology

**Figure 8:**
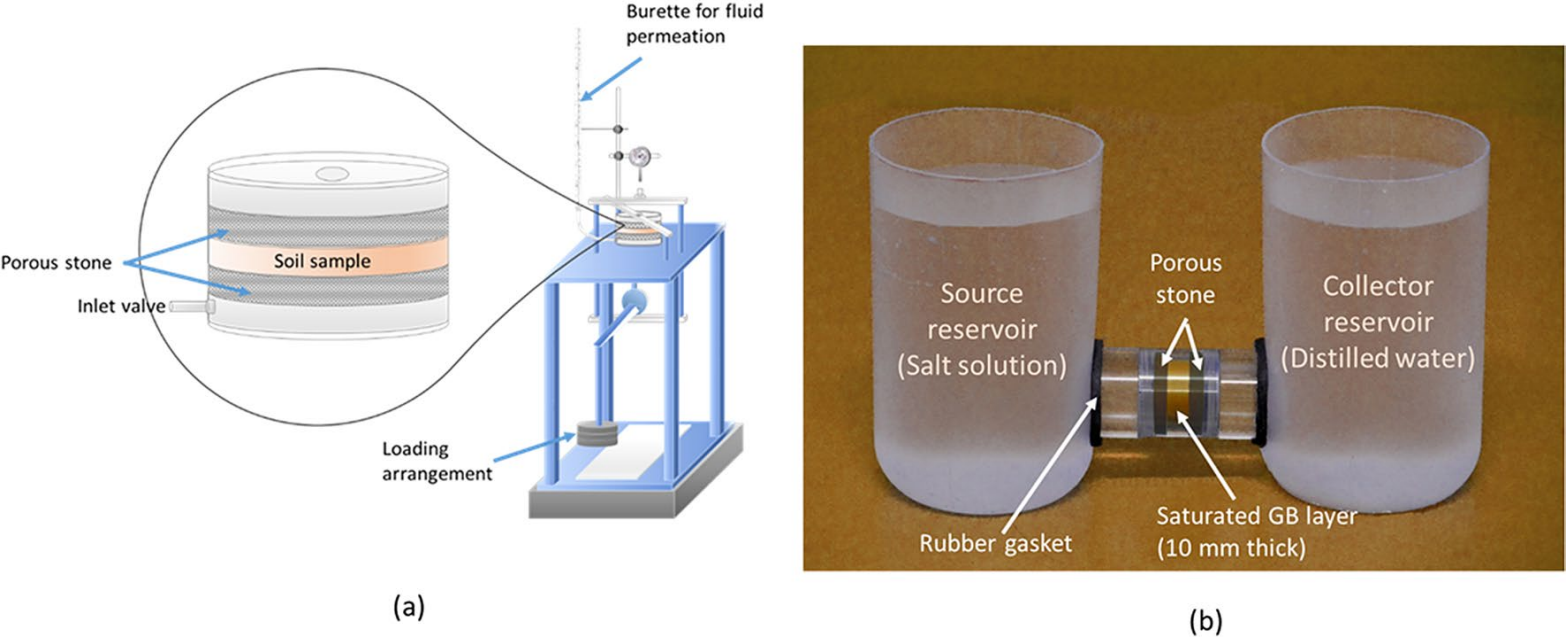
(**a**) Experimental set up for evaluating the sealing and volume change behaviour of compacted soil in the pore-fluid environment, (**b**) through-diffusion set up.

The sealing and swelling ability of the compacted specimens under various landfill chemo-mechanical loading conditions were studied in the permeameter cells. The cell set up for evaluating the volume change and sealing behaviour of the compacted GB under pore-fluid permeation was shown in Fig. 8a. The cell was fabricated from a solid perspex tube to accommodate 10 mm thick and 54 mm diameter specimen with two movable porous stones on either end. The placement density of GB within the GCL was ~ 1.17 Mg/m^3^, which was similar to the estimated placement density in many GCLs^31,55^. The GB was thus compacted in the cell at a dry density of 1.2 Mg/m^3^ to simulate the field conditions. Filter papers were placed between the porous stone and GB on either side to prevent any clogging of the porous stones by clay during the permeation experiments. The cell was then carefully positioned on the loading assembly. The cell was connected to a graduated burette with a valve on one end, which facilitates the permeation of pore-fluid through the compacted GB. The burette was filled with the desired pore-fluid of a particular concentration, with the valve closed before the commencement of the test. A strain gauge having a precision of 0.001 mm was fixed firmly on the top of the sample to record the vertical displacement of the compacted soil sample, as shown in Fig. 8a. The test was initiated by maintaining the desired mechanical stress on the sample and, consequently, allowing the permeation of the targeted fluid from the bottom of the cell by opening the valve of the burette. All the tests were performed at two mechanical stresses, viz., 50 kPa and 100 kPa, to understand the swelling and sealing potential of the clay specimen under the field conditions. The fluid permeation rate (i.e., hydraulic conductivity) was estimated by the falling head technique^56^ whilst the compacted specimen was permeated with the desired pore-fluid under the applied stresses. The test was continued until the fluid permeation rate was constant over a period of 24 h. The vertical displacement reading (expressed as normalized thickness, *h/h*_0_) from the strain-gauge and the fluid permeation rate (expressed in m/s) provided the swelling potential and sealing potential of the soil, respectively.

The compacted GB was assumed to have achieved complete sealing by closing all the inter-granular and inter-aggregate pores when a fluid permeation rate achieved the limiting hydraulic conductivity (i.e., 1 × 10^–9^ m/s)^57,58^. Thus, the sealing time for the GB under the influence of different pore-fluids was established based on the permeation rate of the fluid. The volume change behaviour and sealing potential of the kaolin-GB layered system were also established by following the same procedure as adopted for the GB layer alone. However, a 5 mm thick compacted kaolin at 1.7 Mg/m^3^ was placed below the compacted 5 mm thick GB layer to allow salt permeation from the bottom to reach the kaolin layer in the beginning. As the sealing ability of the GB is not influenced by its thickness, an overall 10 mm thick kaolin-GB layer was maintained in the cell.

The laboratory through-diffusion test was performed for estimating the diffusion characteristics of compacted GB, kaolin, and the kaolin-GB layered system in the presence of high ionic-strength salt solution (i.e., 0.5 M KCl). A diffusion cell of 24 mm diameter and 10 mm thickness was fabricated from a solid perspex tube to accommodate the clay plug, as shown in Fig. 8b. The clay samples were statically compacted at dry-state in the diffusion cell, and porous stones were placed on either side of the sample. Rubber gaskets were attached to the cell for the prevention of any leakage. The compaction densities of the clay samples were maintained the same as that of the permeation test, discussed in the previous section. The diffusion cell was carefully connected to the source and collector reservoirs on either end. The reservoirs were then filled with distilled water, and the compacted sample was allowed to saturate from both ends. After ensuring complete saturation of the clay sample, the diffusion experiment was commenced by replacing the source reservoir with the desired salt concentration, and the collector reservoir was also replaced with fresh distilled water. The concentration of the salt in the source and collector reservoirs was monitored periodically for the diffusion analysis.

### Analysis of diffusion tests

The diffusion of salt solutions through compacted clay specimens is theoretically studied using Fick's diffusion equation. The governing differential equation describing 1-D solute transport through saturated soil is given by^59,60^:

1 $$\frac{\partial c}{{\partial t}} = \frac{{D_{e} }}{{R_{d} }}\frac{{\partial^{2} c}}{{\partial x^{2} }}$$

where *D*_*e*_ and *R*_*d*_ are important landfill liner design parameters, referred to as the effective diffusion coefficient and retardation factor, respectively, *x* is the spatial distance from the source, *t* is the time of diffusion, *c* is the solute concentration in the soil pores, and *n* is the soil porosity. The estimation of the design parameters is vital for understanding the diffusion characteristics of the compacted soil under the salt environment.

The mathematical description of initial and boundary conditions for the through-diffusion experiment is found elsewhere^59,61^. Equation (1) along with the initial and boundary conditions were solved to obtain a closed-form analytical solution^61^. The liner design parameters were estimated by minimizing the error between theoretical and experimental concentration data in the source and collector reservoirs at various time intervals by solving the forward analysis using a different set of parameters^40,41,59^. The design parameters for the GB, kaolin, and kaolin-GB layered system under an extreme saline environment were obtained by inputting the experimental measurements.

## Conclusions

The chemo-mechanical behaviour of compacted clays for the landfill application provides the following conclusions:

1. The GB poorly performs due to exposure to high ionic strength solutions of KCl and CaCl_2_ in sealing and developing swelling potential under different mechanical stresses. The increase in the ionic strength of calcium and potassium salts decreases the osmotic pressure between the hydrated particles and fails to overcome the van der Waals forces for complete disintegration of the granules into individual particles. The applied stress improved GB's sealing ability in high ionic strength KCl solution but at the cost of increasing the mechanical collapse. The increased mechanical load helps disintegrate the interparticle bond but collapses due to the reduction in GB’s swelling pressure.
2. The proposed kaolin-GB combined layer performs very well even under adverse saline conditions and low overburden stresses. The kaolinite particles at dry state in a densely compacted kaolin form tiny micropores due to strong coulombic and van der Waals attraction. The saturation by high ionic strength salts further narrows the pore sizes by bringing the particles together due to DDL thinning, which helps reach the lower conductivity. Additionally, the reactive termination sites and basal surfaces retain significant contaminant concentrations to allow underlying GB to expose only to lower ionic strength salt concentrations. Thus GB achieves complete sealing and reduces the mechanical collapse in the presence of high ionic strength salts.
3. The estimated effective diffusion coefficient and retardation factors for the kaolin-GB layer are superior to the individual GB under similar conditions, indicating that the proposed barrier system effectively inhibits the leachates in the long run. The proposed natural barrier material helps the Geoenvironmental engineers to contain the high ionic strength leachate salts in the landfills and safeguard the environment.

## Supplementary Information

Supplementary Information 1.
