# Supplementary material for: Kaolin based protective barrier in municipal landfills against adverse chemo-mechanical loadings
Source: Sci Rep. 2021 May 14;11:10354. doi: 10.1038/s41598-021-89787-z (PMC8121884; doi:10.1038/s41598-021-89787-z)
Supplement: Supplementary file 1 — Supplementary Information 1. [file 41598_2021_89787_MOESM1_ESM.pdf]

Supplementary Information

KAOLIN BASED PROTECTIVE BARRIER IN MUNICIPAL LANDFILLS AGAINST ADVERSE CHEMO-MECHANICAL LOADINGS

Partha Das[1] *and Tadikonda Venkata Bharat*[2]

[1] Research Student, Indian Institute of Technology, Guwahati, Guwahati-781039, Email: partha.das@iitg.ac.in

[2] Associate Professor, Indian Institute of Technology, Guwahati, Guwahati-781039, Email: tvb@iitg.ac.in

Corresponding Author

[2] Tadikonda Venkata Bharat

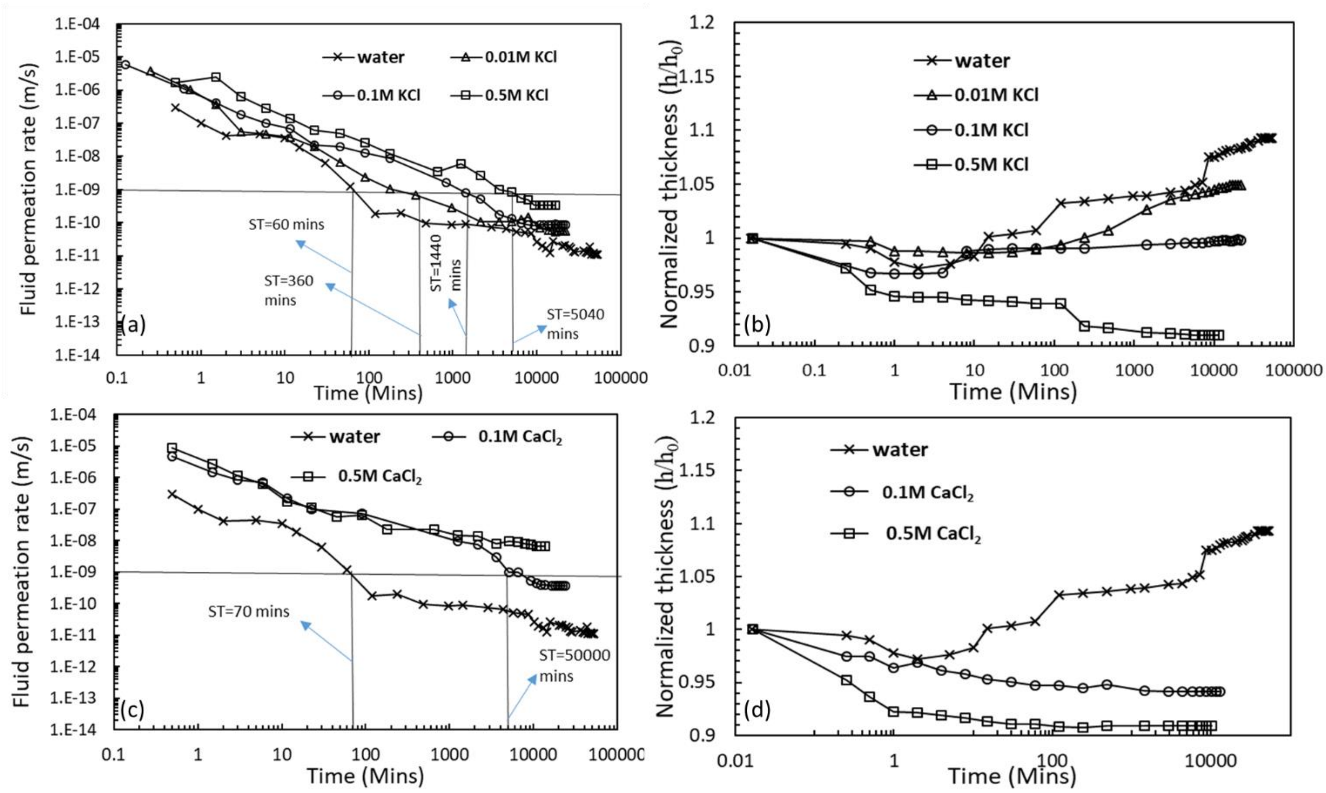

Fig. S1: Temporal variation of (a) fluid permeation rate, and (b) normalized thickness for the GB under applied stress of 100 kPa in the presence of water and various concentrations of KCl; temporal variation of the (a) fluid permeation rate, and (b) normalized thickness of GB under applied stress of 100 kPa in the presence of water and various concentrations of $CaCl_2$
